# ACE-inhibitor/angiotensin receptor blockers (ACE-I/ARBs) therapy in COVID-19 infected dialysis patients

**DOI:** 10.1080/0886022X.2021.1994419

**Published:** 2021-10-27

**Authors:** Ahmed Daoud, Hatem Ali, Vinaya Rao, Vinayak Rohan, Karim Soliman

**Affiliations:** Department of Internal Medicine, Cairo University Kasr Alainy Faculty of Medicine, Cairo, Egypt; Department of Internal Medicine, University Hospitals Coventry and Warwickshire NHS Trust, Internal Medicine, Coventry, United Kingdom of Great Britain and Northern Ireland; Department of Internal Medicine, Medical University of South Carolina, Charleston, SC, USA; Department of Surgery, Medical University of South Carolina, Charleston, SC, USA

Dear Editor,

The novel severe acute respiratory syndrome-coronavirus-2 (SARS-CoV-2) enters human cells by binding to the membrane bound angiotensin converting enzyme-2 (ACE-2) [1]. Angiotensin converting enzyme inhibitor/Angiotensin receptor blockers (ACE-I/ARBs) leads to upregulation of ACE-2 expression. This may hypothetically increase the risk of corona virus disease-19 (COVID-19) infection, severity and mortality [1]. Jarcho et al. demonstrated the results of retrospective studies favoring that ACE-I/ARBs therapy is not associated with higher mortality or worse outcomes in COVID-19 patients [2]. Other studies, have shown that the use of ACE-I/ARBs among hospitalized COVID-19 patients is associated with lower mortality [3].

In this meta-analysis, we summarize the results of large multicenter studies that assessed the safety of ACE-I/ARBs therapy in COVID-19 infected end stage renal disease (ESRD) dialysis patients. We involved the large multicenter studies published from the beginning of 2020 till May 2021. Hsu et al. performed a large multicenter study in the USA between February and June 2020. The study included 7948 dialysis patients; out of which 438 experienced COVID-19 infection. About ¼ of the COVID-19 infected patients died (109/438) [4]. According to the results of Hsu et al., there was no statistically significant association between ACE-I/ARBs therapy and COVID-19 mortality (OR 0.78, 95% CI 0.45–1.33, *p* = 0.4) [4].

Sanchez-Alvarez et al in their multicenter study included around 580 COVID-19 infected dialysis patients in Spain in March and April 2020 [5]. The mortality rate was 26.3%. On multivariate analysis, ARBs therapy was also not associated with higher COVID-19 mortality (OR 0.66, 95% CI 0.38–1.12, *p* = 0.12) [5].

Lano et al conducted a multicenter observational cohort study in France between March and May 2020 [6]. A total of 2336 dialysis patients were enrolled. Out of 129 COVID-19 infected patients 34 died. Interestingly, this study showed a protective effect of ARBs therapy on mortality in COVID-19 infected dialysis patients (OR 0.093, 95% CI 0.005–0.54, *p* = 0.03). However, the authors outlined the limitation of the observational retrospective design of the study [6]. On pooling the data from the 3 aforementioned studies together, we found that ACE-I/ARBs therapy is associated with a protective effect among COVID-19 infected dialysis patients (OR 0.49, 95% CI 0.03–0.94, *p* = 0.007). These findings are demonstrated in Figure 1. A possible interpretation of our results is that patients not on ACE-I/ARBs might have a baseline hypotension with other multiple risk factors including frailty, all attributing to increased mortality. This may be confounded in favor of the use of ACE-I/ARBs in the hypertensive ESRD cohort. A clue to support this hypothesis, is the “apparent” protective effect of hypertension on mortality in COVID-19 infected dialysis patients found by Hsu et al. (OR 0.50, 95% CI 0.26–0.96, *p* = 0.04) [3] and by the ERA CODA study (OR 0.61, 95% CI 042–0.88, *p* < 0.001) [7].

**Figure 1.**
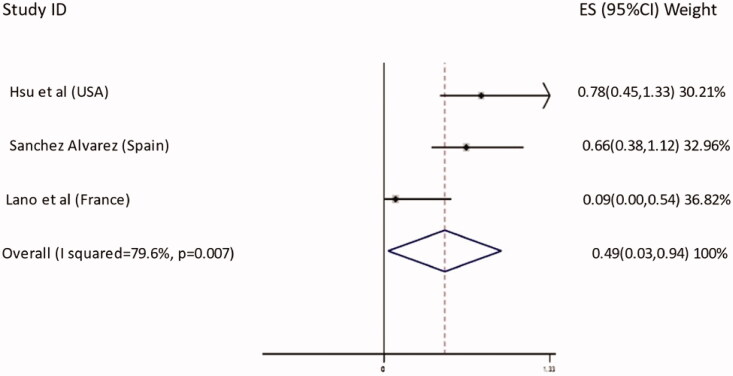
Relationship between ACE-I/ARBs therapy and mortality in COVID-19 infected dialysis patients.

## Conclusion

Our results are in favor of the safe use of ACE-I/ARBs in COVID-19 infected ESRD dialysis patients. A possible protective effect is yet to be determined by further randomized controlled trials assessing the effect of ACE-I/ARBs therapy in COVID-19 infected dialysis patients. It is hard to reach solid conclusions from our meta-analysis due to the small number of studies included due to paucity of literature available.
